# Invasiveness of decompression surgery affects modeled lumbar spine kinetics in patients with degenerative spondylolisthesis

**DOI:** 10.3389/fbioe.2023.1281119

**Published:** 2024-01-08

**Authors:** M. Kosterhon, A. Müller, R. Rockenfeller, A. K. Aiyangar, K. Gruber, F. Ringel, S. R. Kantelhardt

**Affiliations:** ^1^ Department of Neurosurgery, Medical Center of the Johannes Gutenberg–University, Mainz, Germany; ^2^ Institute for Medical Engineering and Information Processing (MTI Mittelrhein), University Koblenz, Koblenz, Germany; ^3^ Mechanical Systems Engineering, Swiss Federal Laboratories for Materials Science and Technology (EMPA), Dübendorf, Switzerland; ^4^ Department of Mathematics and Natural Science, Institute of Sports Science, University Koblenz, Koblenz, Germany; ^5^ Department of Mathematics and Natural Science, Mathematical Institute, University Koblenz, Koblenz, Germany; ^6^ Department of Orthopedic Surgery, University of Pittsburgh, Pittsburgh, PA, United States; ^7^ Faculty of Engineering and Sciences, University of Adolfo Ibanez, Vina del Mar, Chile; ^8^ Faculty of Medicine, University of Bern, Bern, Switzerland

**Keywords:** biomechanics, forward dynamic simulation, spinal stenosis, MBS model, interlaminar fenestration, laminectomy, laminotomy, spondylolisthesis

## Abstract

**Introduction:** The surgical treatment of degenerative spondylolisthesis with accompanying spinal stenosis focuses mainly on decompression of the spinal canal with or without additional fusion by means of a dorsal spondylodesis. Currently, one main decision criterion for additional fusion is the presence of instability in flexion and extension X-rays. In cases of mild and stable spondylolisthesis, the optimal treatment remains a subject of ongoing debate. There exist different opinions on whether performing a fusion directly together with decompression has a potential benefit for patients or constitutes overtreatment. As X-ray images do not provide any information about internal biomechanical forces, computer simulation of individual patients might be a tool to gain a set of new decision criteria for those cases.

**Methods:** To evaluate the biomechanical effects resulting from different decompression techniques, we developed a lumbar spine model using forward dynamic-based multibody simulation (FD_MBS). Preoperative CT data of 15 patients with degenerative spondylolisthesis at the level L4/L5 who underwent spinal decompression were identified retrospectively. Based on the segmented vertebrae, 15 individualized models were built. To establish a reference for comparison, we simulated a standardized flexion movement (intact) for each model. Subsequently, we performed virtual unilateral and bilateral interlaminar fenestration (uILF, bILF) and laminectomy (LAM) by removing the respective ligaments in each model. Afterward, the standardized flexion movement was simulated again for each case and decompression method, allowing us to compare the outcomes with the reference. This comprehensive approach enables us to assess the biomechanical implications of different surgical approaches and gain valuable insights into their effects on lumbar spine functionality.

**Results:** Our findings reveal significant changes in the biomechanics of vertebrae and intervertebral discs (IVDs) as a result of different decompression techniques. As the invasiveness of decompression increases, the moment transmitted on the vertebrae significantly rises, following the sequence intact ➝ uILF ➝ bILF ➝ LAM. Conversely, we observed a reduction in anterior–posterior shear forces within the IVDs at the levels L3/L4 and L4/L5 following LAM.

**Conclusion:** Our findings showed that it was feasible to forecast lumbar spine kinematics after three distinct decompression methods, which might be helpful in future clinical applications.

## Introduction

### Medical background

Back pain resulting from degenerative changes of the spine represents one of the most prevalent medical conditions in the human population (Heliovaara et al., 1989; Schmidt et al., 2007; Raspe, 2012). In addition to conservative therapeutic approaches, surgical intervention plays a crucial role in the management of specific back pain associated with structural skeletal abnormalities (Czabanka et al., 2018). Among these structural abnormalities, degenerative spondylolisthesis (DS) frequently affects the lumbar spine of elderly patients (Pietrantonio et al., 2019). DS involves the anterior displacement of one vertebra relative to the adjacent vertebra, primarily caused by micro-instability within the intervertebral discs (IVDs) and ligamentous structures. The most commonly affected level is L4/L5 (Wiltse et al., 1976). This displacement, coupled with concurrent hypertrophy of intraspinal ligaments, leads to the constriction of the spinal canal and subsequent compression of nerve roots, resulting in spinal stenosis.

### Treatment options for degenerative spondylolisthesis

The main surgical treatment approach is decompression of the spinal canal, employing various techniques, along with the potential addition of a fusion by means of dorsal spondylodesis or minimally invasive approaches to stabilize the affected segment. Decompression of the spinal canal can be achieved through less invasive methods, such as interlaminar fenestration (ILF, also known as laminotomy). During ILF, mostly only the ligamenta flava are removed, either only at the side of the surgical approach or at both sides, mainly performed by undercutting from the side of the approach to the opposite side, while preserving most bony parts (except for the medial aspect of the facet joint). For more extended decompression, a laminectomy (LAM) can be performed, which entails the removal of the whole lamina and, in most cases, also the spinous process and the adjacent ligaments (Benz and Garfin, 2001). Particularly, in cases of mild ventral displacement (Meyerding degree I), an ongoing debate revolves around whether patients solely require decompression or if they could benefit from concurrent stabilization as a primary intervention (Forsth et al., 2016; Ghogawala et al., 2016; Austevoll et al., 2021). To address this question, several radiological signs indicating spinal instability, including ventral displacement of one vertebra in functional X-rays, diminished IVD height, facet joint effusion in MRI, and facet orientation, are commonly employed. However, conflicting results have created ambiguity regarding the ability of these parameters alone to accurately predict the necessity for additional fusion (Stokes and Frymoyer, 1987; Nimmons et al., 2020).

### Biomechanical considerations and computer simulation

One contributing factor to the uncertainty in answering the question of whether only decompression alone or additional fusion should be applied might be the limitation of conventional clinical imaging modalities. CT, MRI, and X-rays can only provide static representations of the spine’s dynamic behavior. Biomechanical aspects, such as altered range of motion (RoM) and the internal forces and moments that occur during movement, cannot be adequately evaluated using these techniques. Nonetheless, *in vitro* studies have already demonstrated the biomechanical effects on the RoM and stiffness of the lumbar spine following various decompression surgeries (Tai et al., 2008; Hartmann et al., 2012; Bisschop et al., 2014; Ho et al., 2015). Computer simulation models offer a valuable means to estimate the internal forces and moments. This might help to objectify therapy decisions, avoiding over- or under-treatment (e.g., sparing instrumentation where not needed and selecting the least destabilizing approach) (Dreischarf et al., 2016).

### Inverse vs. forward dynamic simulation approaches

Extensive development, validation, and utilization of spine and upper body models have been undertaken in numerous studies investigating spinal biomechanics. In the realm of spinal biomechanics, inverse dynamic approaches are commonly employed in *in silico* studies to calculate internal forces and moments. In inverse dynamic simulation, the kinematics of the spinal motion is given in advance, e.g., from motion capturing. Forces and moments are then calculated and optimized to represent the measured kinematic behavior. However, this approach requires both pre- and post-surgical kinematic data, which are not typically available in routine clinical diagnostic procedures, such as X-rays and MRI. An alternative approach is forward dynamic simulation. In this scenario, the simulation starts from a neutral posture, and the transition into another state is induced by the application of forces and moments to the spine. This strategy provides an opportunity to predict alterations in motion resulting from surgical intervention and subsequently compute internal forces and moments. This approach, thus, holds particular value as it could enable the prediction of spinal motion after surgeries and capture both the altered kinematic and dynamic aspects. Due to the complex control of these simulations, only a limited number of studies have adopted a forward dynamic-based simulation approach in investigating spinal mechanics (Silvestros et al., 2019; Guo et al., 2021; Müller et al., 2021; Meszaros-Beller et al., 2023). While some models with neural muscle control have been applied to explore potential therapies, like crouch gait models (Arnold et al., 2006; Steele et al., 2010), the utilization of forward dynamic approaches for lumbar spine models remains relatively scarce. One significant advantage of forward dynamic models lies in their use of six-degree of freedom (DOF) joints for each vertebra, which provide a more realistic representation of spinal movement. In contrast, inverse dynamic approaches require precise kinematic data for each joint, often resulting in the reduction of spinal joints to three DOFs for the entire spine (Christophy et al., 2012).

### Objectives of this study

The aim of this research project was to apply forward dynamic simulations using subject-specific lumbar spine models to assess changes in lumbar spine dynamics resulting from three distinct surgical decompression techniques. Considering the limited availability of dynamic datasets from patients, primarily due to the predominant use of static imaging modalities in clinical settings, the current research heavily relies on outcome studies (Forsth et al., 2016; Ghogawala et al., 2016; Austevoll et al., 2021). Therefore, the application of forward dynamic-based simulations presents a significant opportunity for investigating spinal mechanics. We hypothesize that the flexion–extension moments in IVDs will increase as the decompression technique becomes more invasive.

## Materials and methods

### Building the MBS model of the lumbar spine

A total of 49 patients with degenerative spondylolisthesis and accompanying spinal stenosis, who were treated at the University Medical Center Mainz between January 2017 and July 2018, were identified retrospectively from the departmental information system. The inclusion criteria were monosegmental spondylolisthesis with additional stenosis. Both preoperative CT and preoperative flexion/extension X-ray images had to be available. Only patients with low-grade spondylolisthesis were considered for further evaluation (Meyerding grade I) (Koslosky and Gendelberg, 2020). In this group of patients, the choice of treatment (decompression only or decompression and fusion) is still a matter of ongoing discussion, and only patients eligible for either type of surgery should be included. Therefore, patients with radiological signs of instability (any signs of ventral or dorsal movement in the flexion/extension X-rays compared to the neutral standing X-rays), Meyerding grade >1, findings of spondylolysis, previous spinal surgeries, or fractures were excluded. Of the 49 patients, 18 were considered for further evaluation after applying these inclusion/exclusion criteria. During data preparation, three more patients had to be excluded due to insufficient imaging data (low spatial resolution with slice thickness > 1 mm not sufficient for 3D reconstruction), resulting in a total of 15 patients analyzed. The mean patient age was 67.9 years (*n* = 15; 49–87 years ±10.9 years). The ratio of men to women was 6:9. The study was approved by the local ethics committee, and all subjects had previously provided their consent.

**FIGURE 1:**
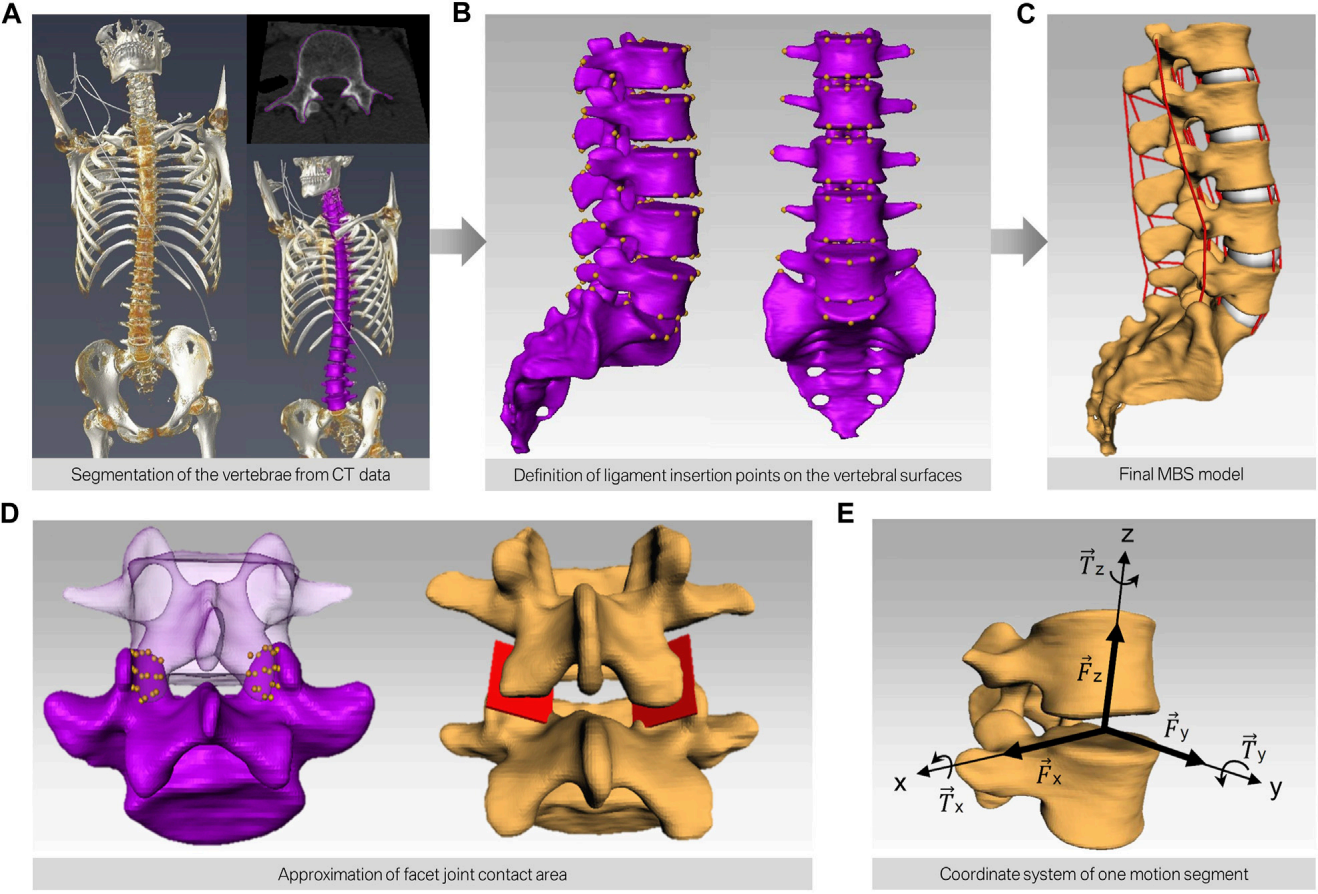
Workflow of building subject-specific MBS models. **(A)** CT scan of human spines and semi-automatic segmentation of the bony structures resulting in a surface model for each lumbar spine (purple). **(B)** Manual definition of the points of interest on the surface of each vertebra. **(C)** Automatic transfer of the bony surfaces and the points of interest to the simulation software. The red lines depict the different ligaments interconnecting each motion segment. **(D)** Modeling of the contact area of the facet joints by defining nine points on each facet and subsequent approximation of a mean plane through these points. **(E)** Alignment of the joint in each motion segment.

**FIGURE 2:**
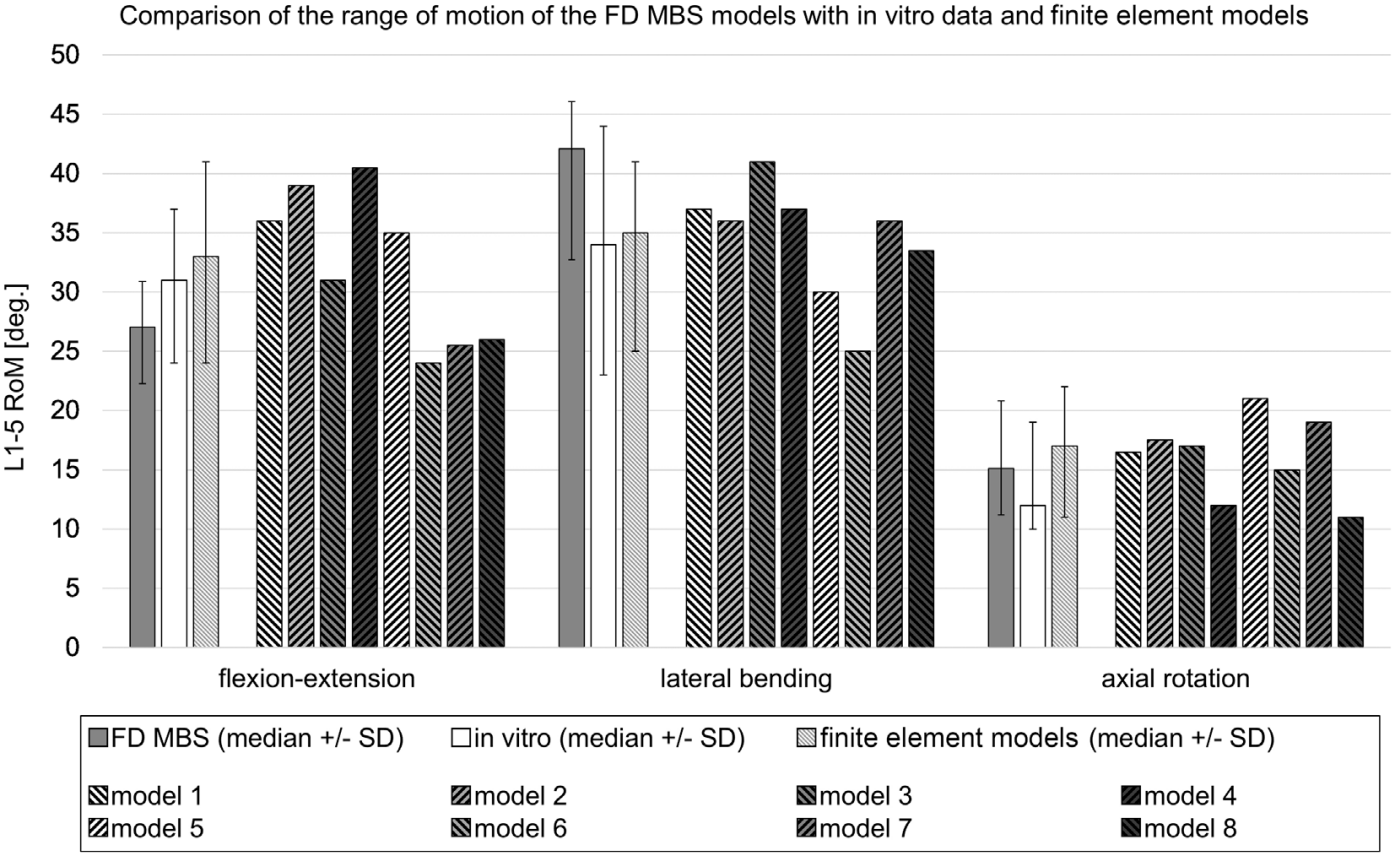
Comparison of the range of motion in forward dynamic-based multibody simulations (FD_MBS) with *in vitro* data and various flexion-extension models taken from the work of Dreischarf et al. (2014). The FD_MBS models, which were subject to the same loading conditions as presented by Dreischarf et al., underwent a consistent 7.5 Nm pure moment at the L1 level in the flexion–extension direction, lateral bending, and axial rotation. To ensure compatibility with the finite element models, the L5 body was fixed in each FD_MBS case. The plain gray bars represent the median RoM ( ± standard deviation) of the 15 FD_MBS models from the current study. The plain white ones show the median RoM ( ± standard deviation) of an *in vitro* study with 10 L1–L5 specimens (Rohlmann et al., 2001). The striped bars show the median RoM ( ± standard deviation) of the eight finite element models as well as the results for each individual finite element model from the work of Dreischarf et al., 2014.

Subsequently, a total of 15 subject-specific lumbar spine forward dynamic-based multibody simulation (FD_MBS) models were built on the basis of these patient CT data. The vertebrae as well as the Os sacrum (SA) were segmented semi-automatically out of CT scans of the 15 subjects in Amira (Thermo Fisher Scientific, Waltham, Massachusetts, United States of America) to preserve the subject-specific curvature, intervertebral disc height, and facet joints (Figure 1A). A total of 47 points of interest per vertebra were set manually on the surfaces according to anatomical landmarks (Figure 1B). The geometries as well as the points of interest were loaded into the simulation tool Simpack (Dassault Systèmes Deutschland GmbH, Munich, Germany). The models consist of six rigid bodies representing the five lumbar vertebrae (L1–L5) and the SA (Figure 1C). To standardize the initial position, the upper endplates of L3 were aligned horizontally in all models, with the SA fixed. The vertebrae were connected by joints with six DOFs and stabilized by passive force elements, e.g., ligaments, IVDs, and facet joints. The ligaments were modeled using a non-linear force-length characteristic and a non-linear rotational characterization for the intervertebral discs obtained from *in silico* experiments (Damm et al., 2020). Translational characteristics of the IVDs’ were defined by viscoelastic elements with corresponding force-elongation characteristics from loading experiments (Schmoelz et al., 2012). Facet joints were realized using contact modeling. Therefore, nine points were defined on each facet joint surface, and a regression plane was calculated (Figure 1D). All passive elements of the model setup were validated in a previous study (Damm et al., 2020) by comparing the intradiscal pressure with literature data from Wilke et al. (1996). For additional validation, the respective range of motion for flexion–extension, lateral bending, and axial rotation was compared to a study by Dreischarf et al., where eight different flexion-extension simulation models were evaluated and compared to biomechanical *in vitro* data (Dreischarf et al., 2014). The presented MBS models were loaded the same way as the models in the study of Dreischarf et al., and forward dynamic-based simulations were performed. This resulted in a RoM for flexion and extension of 22.3°–30.9° (median 27.0°) for the forward dynamic-based multibody simulation (FD_MBS), which lies within the range of the *in vitro* and finite element model measurements reported by Dreischarf et al. Lateral bending and axial rotation results were also within the same order of magnitude (for details, see Figure 2). For further details on the setup, see previous publications (Damm et al., 2020; Rockenfeller et al., 2020; Müller et al., 2021).

### Simulation of three different decompression methods

**FIGURE 3:**
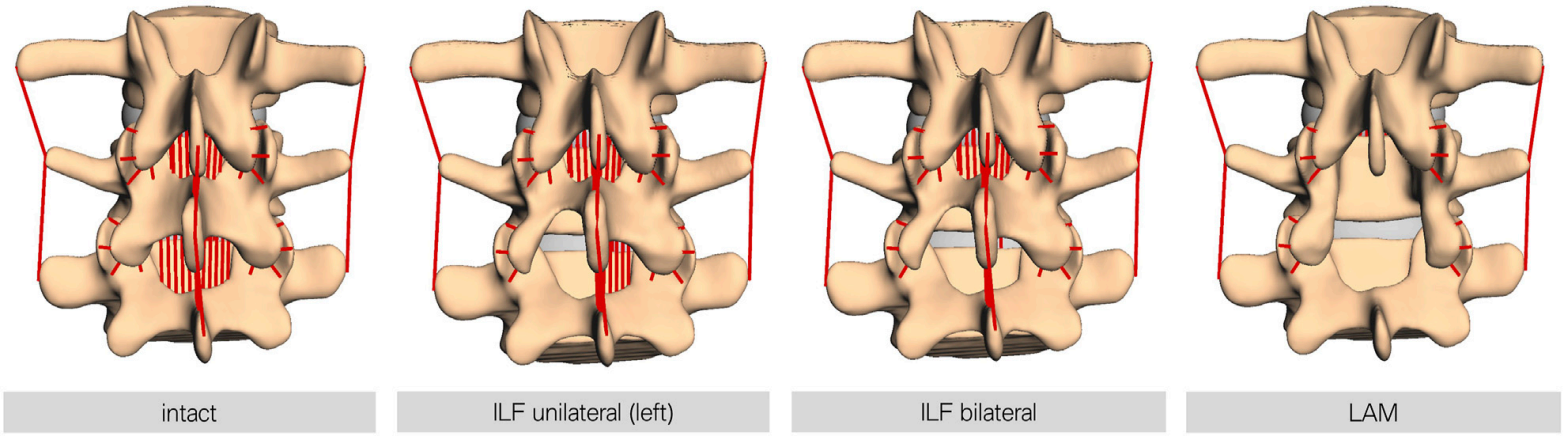
Visualization of the different decompression techniques performed in this study. From left to right, intact: the FD_MBS model in its intact state; ILF unilateral: unilateral interlaminar fenestration—the Ligg. flava at the respective level were removed at the side of fenestration only (left side in all models); ILF bilateral: bilateral interlaminar fenestration—the Ligg. flava at the respective level were removed at the side of fenestration and the contralateral side to mimic the procedure of an undercutting maneuver; and LAM: laminectomy—the Ligg. flava of the respective and the upper level, as well as the interspinous and supraspinous ligaments, were removed. Note: the bony defects here are only for visualization purposes. The bone surface is only relevant for defining the insertion points for ligaments or the contact area of the facet joints and other force elements but otherwise is not necessary for the simulation.

7.5 Nm force was applied to L1, resulting in a ventral flexion of the model (Dreischarf et al., 2014). In addition, each lumbar spine model was preloaded with a constant vertical force of 500 N, representing a generic upper body weight. This generated an additional moment at the fixpoint of the model due to the lever arm of the applied vertical force. The forward dynamic simulation time was set to 3 s. Resulting force data were recorded over the whole movement sequence. Four different modes were investigated: (I) intact, as a reference, (II) unilateral ILF (uILF), (III) bilateral ILF (bILF), and (IV) LAM. To perform the forward dynamic simulations of the theoretical ILF, the ligamenta (Ligg.) flava were virtually removed in the level L4/L5 unilaterally and bilaterally. For the unilateral ILF, the left parts of the ligament flavum were removed. For LAM additionally, the Ligg. intraspinosus and supraspinosus as well as the Ligg. flava to the L3 and L5 vertebrae were removed from the simulation (Figure 3). The bony structures, e.g., interspinous process or parts of the lamina, were not removed as they were only used to define ligament insertion points and contact area of the facet joints but were not part of the simulation itself.

### Statistical analysis

As modeled forces and moments highly depend on individual geometries (Müller et al., 2021), the absolute simulation outputs are hardly comparable. Hence, a normalization procedure is suggested to assess the influence of uILF, bILF, and LAM on flexion–extension (FE) moment, anterior–posterior (AP) shear force, and superior–inferior (SI) compressive force across the spinal levels. All data post-processing was conducted using MATLAB (Mathworks, Version 2022b). The normalization procedure applies to the output values at the end of the forward simulation after 3s. For each individual model and for each spinal level, the output values of the intact model were taken as reference values, corresponding to 100%. The output values of the virtual operated models were normalized to these values. For each spinal level, an unpaired two-sided *t*-test was performed to decide whether the mean of the normalized values was systematically smaller (<100%) or larger (>100%) than the reference value.

Additionally, all time–force or time–moment curves of the 15 subjects were plotted over the simulation time of 3s. A subsequent statistical parametric mapping (SPM from the MATLAB package available at https://spmid.org (Pataky, 2012)), i.e., a paired two-sided *t*-test, was performed on the difference between the intact state and each decompression technique to reveal significant differences in forces and moments over time. A *z*-score above the positive (97.5%-) quantile indicates a significantly higher force/moment in models with the respective decompression method compared to the intact models, while the opposite holds for a *z*-score below the negative (2.5%-) quantile.

## Results

**FIGURE 4:**
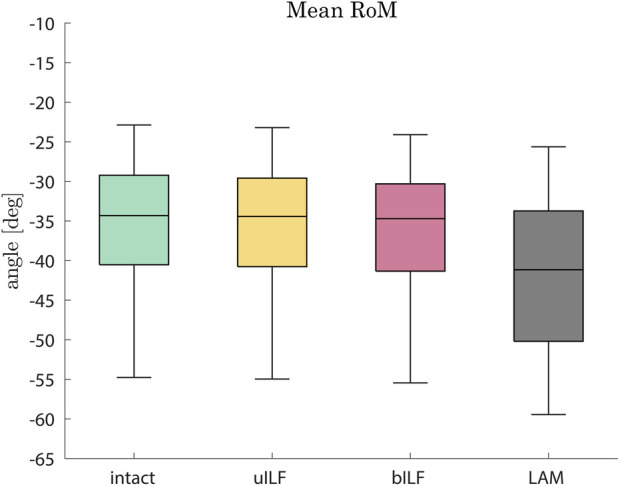
Boxplots of the range of motion in the flexion direction after the full inclination of the FD_MBS for the intact state (green), after virtual uILF (yellow), after virtual bILF (red), and after LAM (black). The RoM was measured at the L1 vertebra at the end of flexion movement.

**FIGURE 5:**
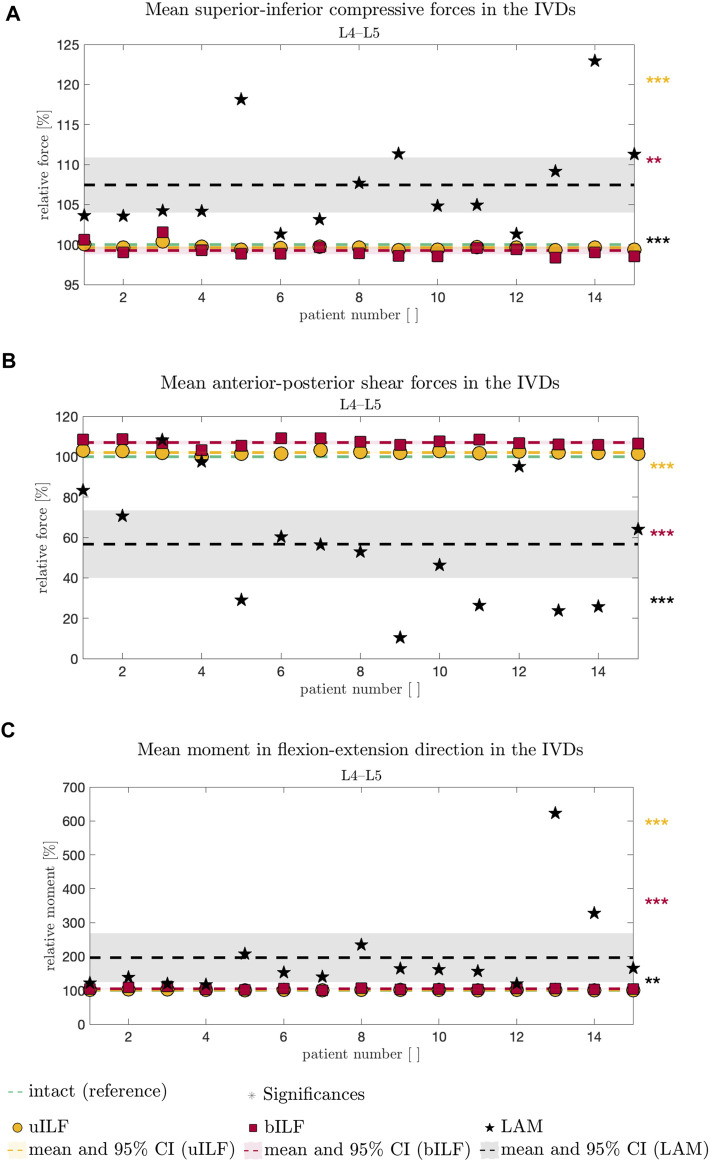
Comparative analysis of SI compressive forces **(A)**, AP shear forces **(B)**, and FE moment **(C)** relative to the intact state (dashed green line in **A–C**) of all 15 FD_MBS models and simulated decompression techniques. The result of the uILF method is represented by the yellow dashed line, and each FD_MBS model is shown as a circle in the corresponding color. The same holds for the bILF method, which is given in red and squares, and the LAM in black and asterisks. The significance of the statistical tests is indicated by alongside asterisks (* = tendency with 0.05 ≤ *p* ≤ 0.1, ** = significant with 0.001 ≤ *p* < 0.05, and *** = highly significant with *p* < 0.001).

**FIGURE 6:**
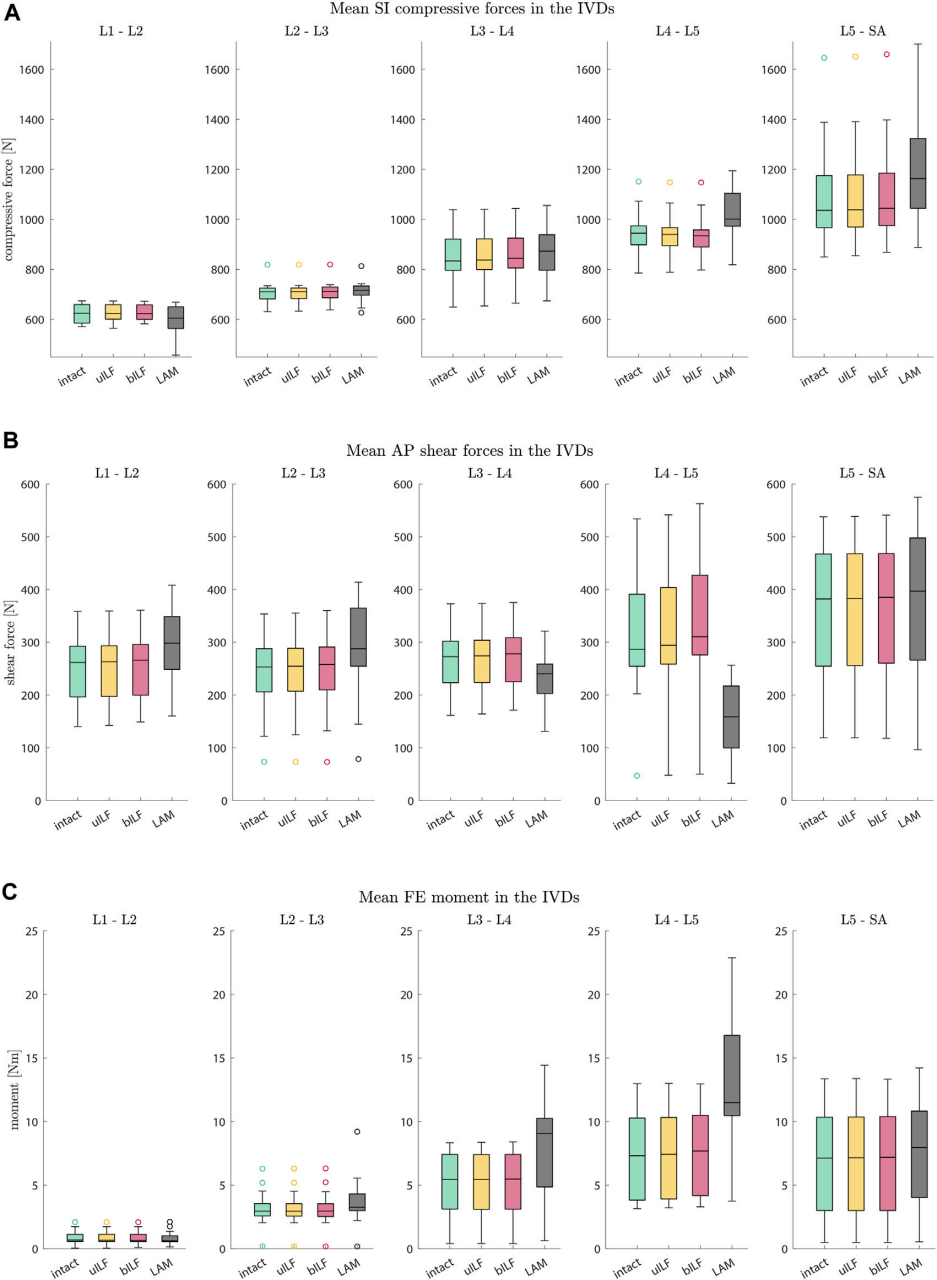
Boxplots of SI compressive forces **(A)**, AP shear forces **(B)**, and FE moments **(C)** for each motion segment at the end of the inclination movement. **(A)** The SI compressive forces at each IVD level increased for more invasive decompression techniques. **(B)** The AP shear forces increased at all levels in the uILF and bILF methods but decreased at levels L3/L4 and L4/L5 following LAM. **(C)** The moments transmitted on the level of the IVD significantly increase with an increase in the invasiveness of decompression, especially in decompressed level L4/L5 (intact → uILF → bILF → LAM).

In the most invasive decompression method (LAM), the range of motion of the FD_MBS increased by 17%, on average (range: 7%–51%). The mean RoM increase was 0.6% (range: 0.3%–1.4%) after uILF and 2% (range: 0.9%–5.3%) after bILF (Figure 4). The more invasive the decompression method, the more the superior–inferior (SI) compressive forces of the IVDs increased. However, this effect was observed more clearly in the decompressed level L4/L5 and its adjacent levels. The anterior–posterior (AP) shear forces increased in the most invasive decompression technique (LAM) in the levels L1/L2, L2/L3, and L5/SA but decreased significantly in the levels L3/L4 and L4/L5. In contrast, the AP shear forces increased at all levels in the uILF and bILF methods. Mean moments transmitted from the upper to the lower vertebrae in the flexion–extension direction increased at all levels when performing uILF, bILF, and LAM. The results of the virtual decompression techniques are shown relative to the intact state in Figure 5 for the level L4/L5 and absolute for all levels in Figure 6.

### Superior–inferior compressive forces in the IVDs

The mean compressive force of the IVDs was calculated in the superior–inferior (SI) direction.

At the L4/L5 level, the SI compressive forces decreased after uILF (−0.38%) and bILF (−0.74%) but increased after LAM (7.44%). In the levels L2/L3, L3/L4, and L5/SA, the SI compressive forces increased after all decompressions. In the L1/L2 level, the mean SI compressive force decreased following LAM.

Larger variations were observed after virtually performing LAM than in the less invasive methods, e.g., uILF: -0.7%–0.41%, bILF: -1.62%–1.57%, and LAM: 1.32%–22.94% for the L4/L5 level (see Figures 5, 6).

### Anterior–posterior shear forces

The mean anterior–posterior (AP) shear forces in the IVDs increased in the L1/L2, L2/L3, and L5/SA levels. For more invasive decompression techniques, the increase of AP shear forces was higher (e.g., uILF: 0.56%, bILF: 1.94%, and LAM: 16.1%). In the levels L3/L4 and L4/L5, the AP shear forces exhibit a decrease of −11.82% and −43.3%, respectively, following virtual LAM. Conversely, subsequent to uILF and bILF, an increase in AP shear forces was observed, mirroring the trend observed in the remaining spinal levels.

### Intervertebral FE moments

The mean moments transmitted from the upper to the lower vertebra in the flexion–extension direction increased at all levels following uILF, bILF, and LAM except the level L1/L2. For the L4/L5 level, the FE moments increased by approximately 1.3% and 4.79% in the case of uILF and bILF, respectively. LAM resulted in a substantially higher increase of 96.58% in FE moment. This circumstance was also found in all other segments, especially the adjacent L3/L4 level, as depicted in Figure 6C.

### L4/5 characteristics over time

**FIGURE 7:**
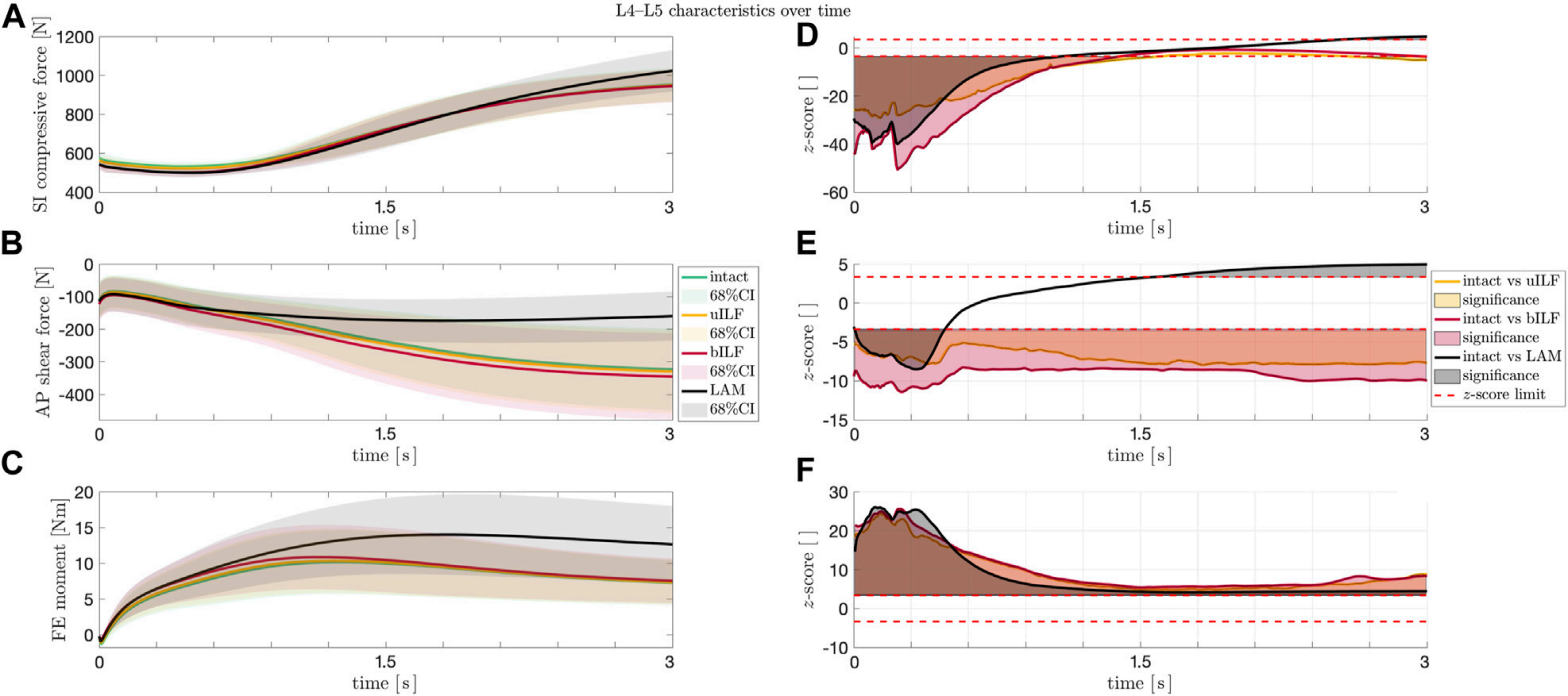
**(A–C)** Solid lines: mean SI compressive force **(A)**, mean AP shear force **(B)**, and mean FE moment **(C)** at the L4/L5 level during the flexion movement. Shaded areas: 68% confidence region (mean plus–minus one standard deviation). The SI compressive forces **(A)** showed an increase at the end of the movement sequence for LAM, whereas the AP shear forces and the FE moments of the IVDs increased significantly in the most invasive decompression technique (LAM). **(D–F)** Paired *t*-test. Solid lines: z-score for SI compressive force **(D)**, AP shear force **(E)**, and FE moment **(F)** at the IVD L4/L5 level during the flexion movement for intact vs. uILF (yellow), intact vs. bILF (red), and intact vs. LAM (black). Filled areas **(D–F)**: significances. Dashed line **(D–F)**: the calculated SPM two-tailed *z*-score limits (95%) for SI compressive forces, AP shear forces, and FE moments were ± 3.64, ± 3.40, and ± 3.36, respectively. Values beyond these limits suggest a significant difference between the forces/moments acting after the corresponding operation method as compared to the intact case.

Additionally, the SI compressive forces, AP shear forces, and FE moments were not only analyzed at the end of the flexion but also monitored throughout the whole course of the motion. As depicted in Figure 7A and Figure 7D, the SI compressive forces were significantly lower in the uILF, bILF, and LAM groups compared to the intact state, especially at the beginning of the motion. Additionally, a significant increase in the SI compressive force is observed in the LAM group at the end of the motion (Figure 7D). The mean AP shear forces of the uILF and bILF groups were significantly higher throughout the entire motion. In contrast, significant decreases in the AP shear forces were observed following LAM at the end of the motion (Figure 7B and Figure 7E). The FE moment increased in the uILF, bILF, and LAM groups (Figure 7C and Figure 7F).

For detailed information about all calculated internal forces see Table 1.

**TABLE 1:** Calculated mean values of all 15 FD_MBS lumbar spine models sorted by level and performed virtual decompression surgery. *M*_*FE*_ *:* Intervertebral moment in flexion-extension direction*. F*_*SI*_ *:* Superior-inferior compressive forces in the IVDs. *F*_*AP*_ *:* anterior-posterior shear forces in the IVD. *F*_*PLL*_ *:* posterior longitudinal ligament force. *F*_*CL*_ *:* capsular ligament forces (values for the L1-L2 and L2-L3 level have not been calculated), *not evaluated.

Level	Moment M_FE_ [Nm] ± SD [min - max]				Compressive force F_SI_ [N] ± SD [min - max]				Shear force F_AP_ [N] ± SD [min - max]				Ligament force F_PLL_ [N] ± SD [min - max]			
	Intact	uILF	bILF	LAM	Intact	uILF	bILF	LAM	Intact	uILF	bILF	LAM	Intact	uILF	bILF	LAM
L1-L2	−0.84 ± 0.54 [(−2.09)–0.03]	−0.84 ± 0.54 [(−2.09)–(−0.03]	−0.84 ± 0.53 [(−2.09)–(−0.1)]	−0.84 ± 0.53 [(−2.09)–(−0.14)]	627.48 ± 35.4 [571.15–674.1]	629.0 ± 34.04 [564.06–673.39]	629.04 ± 32.03 [582.61–672.26]	600.59 ± 61.24 [464.72–668.93]	249.45 ± 61.47 [140.16–358.24]	250.68 ± 61.19 [142.51–359.12]	253.68 ± 60.44 [149.06–360.66]	288.42 ± 70.72 [160.18–408.06]	63.31 ± 42.75 [4.37–52.0]	63.62 ± 42.56 [4.37–52.0]	64.14 ± 43.17 [4.36–52.31]	67.8 ± 46.08 [4.35–63.17]
L2-L3	−3.05 ± 1.51 [(−6.29)–0.18]	−3.05 ± 1.51 [(−6.3)–0.17]	−3.05 ± 1.52 [(−6.31)–0.17]	−3.62 ± 2.06 [(−9.21)–0.17]	668.68 ± 179.96 [40.35–818.29]	669.6 ± 180.03 [40.81–818.55]	671.69 ± 180.54 [40.51–819.29]	675.58 ± 180.69 [44.49–813.0]	243.79 ± 73.9 [73.39–353.49]	245.24 ± 74.02 [73.14–355.32]	248.91 ± 74.33 [73.15–360.18]	285.35 ± 91.08 [78.64–413.82]	110.6 ± 99.09 [1.54–45.82]	111.64 ± 99.99 [1.55–46.3]	113.78 ± 100.7 [1.55–46.64]	128.3 ± 115.29 [1.51–58.73]
L3-L4	−5.15 ± 2.29 [(−8.33)–(−0.4)]	−5.16 ± 2.3 [(−8.37)–(−0.4)]	−5.18 ± 2.32 [(−8.4)–(−0.4)]	−7.82 ± 3.7 [(−14.42)–(0.63)]	852.59 ± 106.19 [649.14–1038.9]	854.74 ± 105.58 [653.23–1040.3]	859.99 ± 104.15 [664.75–1043.6]	889.0 ± 107.03 [673.77–1055.3]	265.28 ± 58.9 [161.52–372.88]	266.73 ± 58.77 [164.63–373.63]	270.25 ± 58.48 [171.24–375.18]	231.29 ± 46.93 [131.22–321.08]	174.18 ± 171.52 [0.2–128.96]	175.8 ± 171.74 [0. 21–129.26]	179.66 ± 173.16 [0.21–130.4]	256.5 ± 210.8 [0.37–158.17]
L4-L5	−7.28 ± 3.12 [(−12.99)–(−3.14)]	−7.36 ± 3.12 [(−13.0)–(−3.21)]	−7.57 ± 3.11 [(−12.96)–(−3.29)]	−12.66 ± 5.35 [(−22.87)–(−3.74)]	953.07 ± 85.92 [784.89–1150.8]	949.4 ± 84.74 [788.08–1147.9]	945.78 ± 83.43 [797.2–1147.2]	1023.9 ± 106.28 [818.13–1194.0]	322.59 ± 125.48 [46.94–533.91]	329.37 ± 127.95 [47.9–541.57]	345.02 ± 132.95 [50.11–562.69]	159.83 ± 75.79 [32.74–282.91]	101.48 ± 81.48 [9.06–39.1]	105.37 ± 85.41 [9.33–39.61]	115.34 ± 96.82 [9.94–42.19]	160.69 ± 106.53 [9.34–179.78]
L5-SA	−6.69 ± 4.15 [(−13.36)–(−0.49)]	−6.69 ± 4.15 [(−13.38)–(−0.49)]	−6.7 ± 4.15 [(−13.33)–(−0.48)]	−7.61 ± 4.61 [(−14.23)–(−0.54)]	1096.3 ± 211.61 [549.62–1645.8]	1099.6 ± 211.56 [854.56–1650.3]	1107.6 ± 211.28 [868.13–1659.8]	1173.1 ± 218.11 [887.58–1701.0]	350.57 ±142.15 [118.91–537.73]	351.24 ± 142.59 [118.9–538.59]	352.75 ± 143.82 [117.96–540.9]	359.25 ± 156.3 [96.36–574.82]	280.47 ± 373.37 [3.35–141.99]	281.94 ± 374.36 [3.39–142.39]	285.34 ± 377.47 [3.48–143.66]	310.61 ± 401.9 [3.63–150.33]

Level	**Ligament force F**_**CL**_ **[N] ± SD; [min - max]**															
	Intact				uILF				bILF				LAM			
L1-L2	*				*				*				*			
L2-L3	*				*				*				*			
L3-L4	left: 170.77 ±115.7; [76.65 – 548.46] right: 139.02 ±84.25; [14.71 – 314.45]				left: 171.12 ± 116.04; [76.86–550.96] right: 140.44 ± 84.6; [14.98–316.31]				left: 174.78 ± 116.19; [78.51–554.91] right: 142.06 ± 84.67; [14.85–318.17]				left: 230.14 ± 130.51; [131.17–665.66] right: 188.6 ± 95.13; [18.68–355.92]			
L4-L5	left: 180.46 ±72.22; [20.57 – 285.77] right: 180.56 ±90.93; [65.74 – 414.35]				left: 185.48 ± 73.59; [20.94–290.09] right: 183.01 ± 91.35; [66.8–417.5]				left: 193.93 ± 76.74; [21.56–299.61] right: 193.57 ±93.39; [71.06–430.1]				left: 271.5 ± 87.34; [77.99–425.63] right: 292.4 ±120.67; [117.47–531.05]			
L5-SA	left: 162.15 ±129.86; [15.23 – 443.57] right: 180.45 ±158.17; [2.59 – 565.65]				left: 162.35 ±130.09; [15.32–444.97] right: 181.57 ±158.65; [2.71–568.2]				left: 164.68 ± 130.77; [17.21–448.57] right: 182.48 ± 159.56; [2.65–572.09]				left: 177.21 ± 133.35; [19.08–467.39] right: 194.15 ± 166.15; [2.64–594.54]			

## Discussion

### Discussion of simulation results

The most prominent alterations were observed in the moments transmitted on the IVDs. Surprisingly, the AP shear forces following LAM decreased significantly at levels L3/L4 and L4/L5 and increased in the other levels. Greater disparities in the results were observed in the AP shear forces of the LAM simulations. The uILF and bILF groups showed an increase in AP shear forces in all levels and fewer variations between the different subject-specific FD_MBS. The SI compressive forces increased in all levels except the first level, L1/L2, after virtual decompression. Similar to the AP shear force, results showed the SI compressive forces to have larger disparities between the spines in the LAM group.

Similar findings were also shown with biomechanical measurements by Cunningham et al., who observed increasing intradiscal pressures (IDP) following resection of dorsal structures (Cunningham et al., 1997). In accordance with the presented observations, studies conducted by Rao et al. demonstrated an increase in IDP in the anterior region and a decrease in the posterior region of the disc after ILF and LAM *in vitro* (Rao et al., 2002). These findings further support the observation of increased FE moments after the removal of dorsal structures.

*In vitro* studies already showed significant alterations of the RoM following a LAM (Hartmann et al., 2012; Bisschop et al., 2014), whereas there is no significant difference between an intact lumbar spine and the spine after uILF and bILF (Tai et al., 2008; Ho et al., 2015).

However, the results presented in this study demonstrate that the more invasive the decompression surgery is, the greater the change in internal FE moments. It is essential to emphasize that considering only the mean values of all the measurement data might be misleading. The human spine exhibits a great deal of individuality, including variations in curvature, which leads to diverse internal forces and moments (Müller et al., 2021). To address this variability, we conducted a paired *t*-test (see Materials and methods, Figure 7) to account for the individual differences among patients. These findings also highlight the importance of incorporating patient-specific characteristics in spine modeling and kinematic measurements (Dombrowski et al., 2018). Such an approach is vital for accurately representing the biomechanics of the spine and its response to surgical interventions.

Overall, the present study shows t/hat SI compressive forces and FE moment increase with an increase in invasiveness of the decompression surgeries (uILF → bILF → LAM). Interestingly, Berger-Roscher et al. showed in *in vitro* experiments that a combination of flexions and rotational moments resulted in more intradiscal lesions when compared to compressive forces alone (Berger-Roscher et al., 2017). Considering this, along with the presented observation of significantly increased FE moments, it is plausible that this phenomenon could contribute to the accelerated degeneration of decompressed and adjacent segments. However, no data are currently available to predict when these changes will manifest as clinically relevant symptoms and whether proactive additional stabilization might be necessary. Surprisingly, the AP shear forces decreased in levels L3/L4 and L4/L5 following LAM. At this point, it must be emphasized that we cannot say how significant the influence of the musculature is on the shear forces after a LAM. However, it can be assumed that the more invasive the operation is, the more unstable the lumbar spine becomes. The hypothesis that FE moments increase with an increase in invasiveness can be confirmed. Accordingly, we assume a potential correlation between FE moments and unstable spines. This assumption is supported by large disparities in the results following LAM. However, it is important to keep in mind that due to the small sample size, selection bias might have occurred.

### Interpretation of the results from a clinical point of view

There exist several techniques for surgical decompression of the spinal canal. Over decades, the more invasive variant of LAM was considered to be the gold standard in spinal decompression surgery in many countries, and it is still the same at present (Overdevest et al., 2015). Nevertheless, more and more minimally invasive techniques are used and reported in the literature, such as uILF and bILF (often also described as unilateral and bilateral laminotomy) (Schär et al., 2019). They retain midline structures, cause less muscular trauma, and preserve more bony structures (Overdevest et al., 2015). While there is no universally applicable answer regarding the superior technique, some have identified potential benefits of less invasive procedures. These may include reduced postoperative pain, shorter hospital stays, and a decreased likelihood of iatrogenic instabilities. (Mobbs et al., 2014; Overdevest et al., 2015). Keeping in mind that LAM is still an option in some cases, this study opted to investigate the biomechanical effects of these three widely used techniques (unilateral ILF, bilateral ILF, and LAM).

Decompression surgery is a complex task, and removal of the ligaments is only an approximation to the complexity of real surgical interventions. In many cases, surgeons would also remove the medial aspects of hypertrophic facet joints in order to decompress the lateral recess (Mobbs et al., 2014). In the presented model, the bony surface of the vertebrae was used only to define the insertion points of the ligaments, the IVD area, and the contact plane of the facet joints. Hence, the bone was only used to setup the correct spacing between these elements. The bony structures themselves were not necessary for our simulation and, therefore, were not used for calculating the internal forces.

The model did not investigate the amount of decompression achievable by the different decompression techniques, which is crucial for short-term patient outcomes, such as relief of leg pain. Another important factor for clinical patient outcome is the degree of foraminal stenosis, which also has to be considered and thoroughly decompressed. Instead, the study focused on biomechanical effects resulting from the removal of more or less passive structures (ligaments). It was demonstrated that LAM showed significantly greater changes in internal forces than other methods of decompression. This in turn might cause new instability and may influence the long-term outcome of patients by means of renewed back pain after an initial period of being pain-free. A systematic review by Overdevest et al. analyzed four high-quality randomized controlled trials (RCTs) and six low-quality RCTs to compare unilateral/bilateral laminotomy and LAM (Overdevest et al., 2015). One major finding was that iatrogenic instability occurred more often after LAM than after the other decompression method. This instability results in renewed back pain and often additional fusion surgery. Assuming that this secondary instability arises from increasing internal forces, such as compression or rotational moments, our simulation model could support this observation and may favor minimally invasive techniques, such as ILF, over LAM. Due to the known limitations of simulations as well as the small sample size, these findings must not be overinterpreted and cannot give general applicable medical advice.

### The potential added value of forward dynamic computer simulation models

Mainly in the treatment of mild degenerative spondylolisthesis, there is still an ongoing debate on whether patients benefit from additional stabilization in the first place or not (Forsth et al., 2016; Ghogawala et al., 2016; Austevoll et al., 2021). Ghogawala et al. investigated the differences between decompression only *versus* decompression and fusion surgery (Ghogawala et al., 2016). Within a follow-up period of 4 years, they found a significantly higher amount of reoperations necessitating secondary fusion due to secondary instability in the group that received decompression only (34% vs. 14%). All patients received LAM for decompression. Our findings of increased compressive forces and rotational moments after LAM could be one factor for increasing instability over time and may support this observation. One-third of patients in the aforementioned study received reoperation. The study by Ghogawala et al. and a recent meta-analysis by Gadjradj et al. state that until now, there are no clear predictors to distinguish between patients who need decompression only vs. those needing additional fusion (Ghogawala et al., 2016; Gadjradj et al., 2023).

Nonetheless, several radiological indicators of spinal instability, such as sliding in functional X-rays, decreased height of the IVD, facet joint effusion, and orientation of the facets, are widely used to answer this question (Park et al., 2004; Chun et al., 2015; Heo et al., 2015; Strube et al., 2019). However, there are contrary findings which make it unclear whether these parameters alone can predict the need for additional fusion:

A survey among German spine surgeons showed that >92% see hypermobility, mostly measured in functional X-rays, as a reason to perform additional fusion (Strube et al., 2019). In addition to the fact that these X-rays are highly dependent on patient compliance and the ability to flex forward (Hayes et al., 1989), others have found them to underestimate hypermobility (Dombrowski et al., 2018) or not be useful for diagnosing lumbar instability (Stokes and Frymoyer, 1987).

In the case of facet joint effusion, there is a strong correlation between such a condition in MRI and ventral sliding in functional X-rays (Chaput et al., 2007; Rihn et al., 2007), but others could not find a significant difference in patient outcome when used as a criterion for adding fusion to decompression alone (Lattig et al., 2015).

A common feature among the abovementioned diagnostic tools, such as CT, MRI, and X-rays, is that they only statically depict the dynamically complex behavior of the spine in a snapshot. Dynamic investigation methods (Aiyangar et al., 2014; Dombrowski et al., 2018; Wawrose et al., 2020; Aiyangar et al., 2023) that can provide individual functional information could improve the decision-making process but are difficult to assess and integrate into clinical routine.

Computer simulation models could help gain more insights and may close this diagnostic gap in the future and help include individual dynamic biomechanical information. Especially in the modeling of surgical methods, the use of individual models seems to be important. In the case of degenerative spondylolisthesis, the simulation data showed a significant increase of SI compressive forces and FE moments, as well as a significant reduction of AP shear forces in levels L3/L4 and L4/L5 following LAM. The simulated LAM shows the most significant results. The significant results become evident when considering the mean values as well as by the paired *t*-test. Although these facts were observed in all patients, the results strongly differed among the individuals, especially after a LAM.

This fact might be a potential candidate to act as a future decision criterion: patients who show only a small amount of FE moments after simulated decompression might be treated with decompression only, whereas patients who show a high amount of increased FE moments might be better treated with decompression and additional fusion. This could help avoid overtreatment on the one side but, on the other side, prevent those who will develop instability over time from having a secondary fusion surgery. Furthermore, this might reduce overall costs for the medical system. However, the practical relevance and clinical significance of this hypothesis have to be investigated in further studies with a larger patient collective and long-term clinical follow-up data. This might allow validating the observations in real-world scenarios.

It can be summarized that further development and application of subject-specific forward dynamic simulation models hold promise in enhancing our understanding of spinal biomechanics, aiding in the prediction of post-surgical outcomes, and enabling more informed and personalized therapeutic decisions for patients.

## Limitations and perspective

Computer simulation models have demonstrated the potential to enhance the empirical basis for decision-making processes by allowing the assessment of biomechanical effects associated with various surgical procedures. However, it is important to emphasize the preliminary nature of findings from computer simulation models. This also applies to the presented models, which have certain limitations. These include the absence of muscles, as well as the exclusion of the thoracic spine, ribcage, and cervical spine. These limitations restrict the comprehensive evaluation of biomechanics in the models. At this point, it must be emphasized that the main objective of this study was to apply the forward dynamic approach to predict changes in spinal motion after a simulated surgical procedure. Given the inherent complexity of the forward dynamic approach, especially compared to the commonly used inverse approach, our first step is to use an “*in-vitro*” or “*ex vivo*” model that serves as a representation of a cadaveric setup. As mentioned in Introduction, in the inverse dynamic approach, the DOF of joints often have to be reduced to gain stable simulation results. Here, we present a forward dynamic model without restricting the DOF of the joints. Contrary to inverse dynamics, the use of pure forward dynamic simulations provides a significant advantage in predicting spinal motion, particularly after surgeries have been performed. It is important to note that the majority of commonly used models are employed in inverse dynamic simulations or static analysis of human motions. These approaches require precise kinematic data for each joint. In spinal biomechanics analysis, there is a dearth of research employing the forward dynamic approach, with only limited studies exploring its potential (Rupp et al., 2015; Silvestros et al., 2019; Guo et al., 2021; Müller et al., 2021; Meszaros-Beller et al., 2023). Conversely, this approach is relatively more widely employed in gait analysis.

Paraspinal muscles, such as the multifidus muscle, have been shown to be of great importance in spinal stabilization. Muscle simulation studies showed, e.g., decreasing pressure values in the IVDs with active multifidus vs. inactive or the absence of muscles (Wang et al., 2023). On the other hand, muscle simulation entails a whole set of new challenges, such as differences in training status and the unknown extent of muscle activation. To especially address the latter task, different approaches have been proposed, e.g., to optimize muscle activation to reach an equilibrium state or to minimize intramuscular pressure (El Bojairami and Driscoll, 2022). Although paraspinal muscles have a huge impact on the biomechanics of the spine, they are not irreversibly altered after surgery. Active structures have the potential to nearly fully recover and can be actively influenced further by training, whereas passive structures, such as ligaments, cannot. Nevertheless, it is recommended for future studies to incorporate models that encompass musculature and the upper body to enable a comprehensive evaluation of biomechanical dynamics in spinal surgeries. This inclusion enables the simulation of active forces necessary for lumbar spine stabilization. Additionally, addressing the simplifications made in the current study, such as utilizing generic characteristics for passive elements (IVDs and ligaments) and applying a predefined load for all simulation models instead of considering individual patient body weights, is necessary. It also remains to be investigated whether the time instant (t = 3s) or mode (intact model as baseline) of the normalization procedure has an influence on the significance statements made above. Yet, given the corresponding consistent results from SPM, this is not to be expected. To mitigate potential confounding variables, the study focused solely on the effects of different decompression techniques on the internal forces and moments of the simulated lumbar spines. Furthermore, a combination of inverse and forward dynamic simulations is advised. Inverse dynamic simulations rely on meaningful kinematic data, and the use of dynamic biplanar stereo X-ray images is recommended to ensure sufficient kinematic analysis of potential destabilizing effects following decompression surgeries (Aiyangar et al., 2014). By integrating the inverse and forward dynamic approaches, along with the corresponding data, more accurate predictions can be made regarding possible destabilization resulting from decompression methods and the subsequent need for fusion. This might be realized by using kinematic data from precise dynamic biplanar X-ray images for the inverse dynamic simulation of the lumbar spine. These outcomes would then be used to fine-tune and calibrate a forward dynamic model configured identically. Subsequently, the computed forces (or muscle activation, stimulation) would be used as inputs for executing forward dynamic simulations, enabling the prediction of altered kinematics following a simulated surgical intervention.

## Conclusion

In conclusion, the FD_MBS models utilized in this study, despite their simplifications, yielded reproducible and plausible results that fell within the range of cadaveric biomechanical studies and finite element models. The utilization of complex forward dynamic simulations presented an opportunity to predict spinal motions following altered configurations. Consequently, it was feasible to forecast the kinematics of human lumbar spines after three distinct decompression methods and calculate the internal forces and moments acting on the passive elements. The investigated decompression methods showed to result in increasing SI compressive forces as well as increasing FE moments the more invasive the chosen method was. These kinetic changes also affected adjacent levels. The shift in forces and moments may serve as a contributing factor in accelerated degeneration processes. Overall, the findings highlight the potential of subject-specific MBS models and forward dynamic simulations in elucidating the biomechanical implications of different decompression methods. Future research should aim to refine and expand upon these models to enhance their accuracy and capture a broader spectrum of clinical scenarios, ultimately providing valuable insights for optimizing surgical interventions and mitigating adverse effects on spinal health.

## Data Availability

The original contributions presented in the study are included in the article; further inquiries can be directed to the corresponding author.

## References

[B1] Aiyangar A. Gale T. Magherhi S. Anderst W. (2023). How many trials are needed to estimate typical lumbar movement patterns during dynamic X-ray imaging? J. biomechanical Eng. 145 (7), 074503. 10.1115/1.4062117 36905174

[B2] Aiyangar A. K. Zheng L. Tashman S. Anderst W. J. Zhang X. (2014). Capturing three-dimensional *in vivo* lumbar intervertebral joint kinematics using dynamic stereo-X-ray imaging. J. biomechanical Eng. 136 (1), 011004. 10.1115/1.4025793 24149991

[B3] Arnold A. S. Liu M. Q. Schwartz M. H. Ounpuu S. Delp S. L. (2006). The role of estimating muscle-tendon lengths and velocities of the hamstrings in the evaluation and treatment of crouch gait. Gait posture 23 (3), 273–281. 10.1016/j.gaitpost.2005.03.003 15964759

[B4] Austevoll I. M. Hermansen E. Fagerland M. W. Storheim K. Brox J. I. Solberg T. (2021). Decompression with or without fusion in degenerative lumbar spondylolisthesis. N. Engl. J. Med. 385 (6), 526–538. 10.1056/nejmoa2100990 34347953

[B5] Benz R. J. Garfin S. R. (2001). Current techniques of decompression of the lumbar spine. Clin. Orthop. Relat. Res. 384 (384), 75–81. 10.1097/00003086-200103000-00010 11249182

[B6] Berger-Roscher N. Casaroli G. Rasche V. Villa T. Galbusera F. Wilke H. J. (2017). Influence of complex loading conditions on intervertebral disc failure. Spine 42 (2), E78–e85. 10.1097/brs.0000000000001699 27187053

[B7] Bisschop A. van Engelen SJPM Kingma I. Holewijn R. M. Stadhouder A. van der Veen A. J. (2014). Single level lumbar laminectomy alters segmental biomechanical behavior without affecting adjacent segments. Clin. Biomech. (Bristol, Avon) 29 (8), 912–917. 10.1016/j.clinbiomech.2014.06.016 25028214

[B8] Chaput C. Padon D. Rush J. Lenehan E. Rahm M. (2007). The significance of increased fluid signal on magnetic resonance imaging in lumbar facets in relationship to degenerative spondylolisthesis. Spine 32 (17), 1883–1887. 10.1097/brs.0b013e318113271a 17762297

[B9] Christophy M. Faruk Senan N. A. Lotz J. C. O'Reilly O. M. (2012). A musculoskeletal model for the lumbar spine. Biomechanics Model. Mechanobiol. 11 (1-2), 19–34. 10.1007/s10237-011-0290-6 21318374

[B10] Chun D. S. Baker K. C. Hsu W. K. (2015). Lumbar pseudarthrosis: a review of current diagnosis and treatment. Neurosurg. Focus 39 (4), E10. 10.3171/2015.7.focus15292 26424334

[B11] Cunningham B. W. Kotani Y. McNulty P. S. Cappuccino A. McAfee P. C. (1997). The effect of spinal destabilization and instrumentation on lumbar intradiscal pressure: an *in vitro* biomechanical analysis. Spine 22 (22), 2655–2663. 10.1097/00007632-199711150-00014 9399452

[B12] Czabanka M. Thomé C. Ringel F. Meyer B. Eicker S.-O. Rohde V. (2018). Operative treatment of degenerative diseases of the lumbar spine. Der Nervenarzt 89 (6), 639–647. 10.1007/s00115-018-0523-3 29679129

[B13] Damm N. Rockenfeller R. Gruber K. (2020). Lumbar spinal ligament characteristics extracted from stepwise reduction experiments allow for preciser modeling than literature data. Biomechanics Model. Mechanobiol. 19 (3), 893–910. 10.1007/s10237-019-01259-6 PMC7203593 31792641

[B14] Dombrowski M. E. Rynearson B. LeVasseur C. Adgate Z. Donaldson W. F. Lee J. Y. (2018). ISSLS PRIZE IN BIOENGINEERING SCIENCE 2018: dynamic imaging of degenerative spondylolisthesis reveals mid-range dynamic lumbar instability not evident on static clinical radiographs. Eur. spine J. 27 (4), 752–762. 10.1007/s00586-018-5489-0 29470715 PMC6032516

[B15] Dreischarf M. Shirazi-Adl A. Arjmand N. Rohlmann A. Schmidt H. (2016). Estimation of loads on human lumbar spine: a review of *in vivo* and computational model studies. J. Biomech. 49 (6), 833–845. 10.1016/j.jbiomech.2015.12.038 26873281

[B16] Dreischarf M. Zander T. Shirazi-Adl A. Puttlitz C. M. Adam C. J. Chen C. S. (2014). Comparison of eight published static finite element models of the intact lumbar spine: predictive power of models improves when combined together. J. Biomech. 47 (8), 1757–1766. 10.1016/j.jbiomech.2014.04.002 24767702

[B17] El Bojairami I. Driscoll M. (2022). Formulation and exploration of novel, intramuscular pressure based, muscle activation strategies in a spine model. Comput. Biol. Med. 146, 105646. 10.1016/j.compbiomed.2022.105646 35751204

[B18] Forsth P. Olafsson G. Carlsson T. Frost A. Borgstrom F. Fritzell P. (2016). A randomized, controlled trial of fusion surgery for lumbar spinal stenosis. N. Engl. J. Med. 374 (15), 1413–1423. 10.1056/nejmoa1513721 27074066

[B19] Gadjradj P. S. Basilious M. Goldberg J. L. Sommer F. Navarro-Ramirez R. Mykolajtchuk C. (2023). Decompression alone versus decompression with fusion in patients with lumbar spinal stenosis with degenerative spondylolisthesis: a systematic review and meta-analysis. Eur. Sect. Cerv. Spine Res. Soc. 32 (3), 1054–1067. 10.1007/s00586-022-07507-1 36609887

[B20] Ghogawala Z. Dziura J. Butler W. E. Dai F. Terrin N. Magge S. N. (2016). Laminectomy plus fusion versus laminectomy alone for lumbar spondylolisthesis. N. Engl. J. Med. 374 (15), 1424–1434. 10.1056/nejmoa1508788 27074067

[B21] Guo J. Guo W. Ren G. (2021). Embodiment of intra-abdominal pressure in a flexible multibody model of the trunk and the spinal unloading effects during static lifting tasks. Biomechanics Model. Mechanobiol. 20 (4), 1599–1626. 10.1007/s10237-021-01465-1 34050846

[B22] Hartmann F. Janssen C. Böhm S. Hely H. Rommens P. M. Gercek E. (2012). Biomechanical effect of graded minimal-invasive decompression procedures on lumbar spinal stability. Archives Orthop. trauma Surg. 132 (9), 1233–1239. 10.1007/s00402-012-1543-2 22592915

[B23] Hayes M. A. Howard T. C. Gruel C. R. Kopta J. A. (1989). Roentgenographic evaluation of lumbar spine flexion-extension in asymptomatic individuals. Spine 14 (3), 327–331. 10.1097/00007632-198903000-00014 2711247

[B24] Heliovaara M. Sievers K. Impivaara O. Maatela J. Knekt P. Makela M. (1989). Descriptive epidemiology and public health aspects of low back pain. Ann. Med. 21 (5), 327–333. 10.3109/07853898909149216 2532521

[B25] Heo Y. Park J. H. Seong H. Y. Lee Y. S. Jeon S. R. Rhim S. C. (2015). Symptomatic adjacent segment degeneration at the L3-4 level after fusion surgery at the L4-5 level: evaluation of the risk factors and 10-year incidence. Eur. spine J. 24 (11), 2474–2480. 10.1007/s00586-015-4188-3 26266771

[B26] Ho Y.-H. Tu Y.-K. Hsiao C.-K. Chang C.-H. (2015). Outcomes after minimally invasive lumbar decompression: a biomechanical comparison of unilateral and bilateral laminotomies. BMC Musculoskelet. Disord. 16, 208. 10.1186/s12891-015-0659-2 26285817 PMC4545783

[B27] Koslosky E. Gendelberg D. (2020). Classification in brief: the meyerding classification system of spondylolisthesis. Clin. Orthop. Relat. Res. 478 (5), 1125–1130. 10.1097/corr.0000000000001153 32282463 PMC7170696

[B28] Lattig F. Fekete T. F. Kleinstück F. S. Porchet F. Jeszenszky D. Mannion A. F. (2015). Lumbar facet joint effusion on MRI as a sign of unstable degenerative spondylolisthesis: should it influence the treatment decision? J. spinal Disord. Tech. 28 (3), 95–100. 10.1097/bsd.0b013e318269c261 22832556

[B29] Meszaros-Beller L. Hammer M. Riede J. M. Pivonka P. Little J. P. Schmitt S. (2023). Effects of geometric individualisation of a human spine model on load sharing: neuro-musculoskeletal simulation reveals significant differences in ligament and muscle contribution. Biomechanics Model. Mechanobiol. 22 (2), 669–694. 10.1007/s10237-022-01673-3 PMC10097810 36602716

[B30] Mobbs R. J. Li J. Sivabalan P. Raley D. Rao P. J. (2014). Outcomes after decompressive laminectomy for lumbar spinal stenosis: comparison between minimally invasive unilateral laminectomy for bilateral decompression and open laminectomy: clinical article. J. Neurosurg. Spine 21 (2), 179–186. 10.3171/2014.4.spine13420 24878273

[B31] Müller A. Rockenfeller R. Damm N. Kosterhon M. Kantelhardt S. R. Aiyangar A. K. (2021). Load distribution in the lumbar spine during modeled compression depends on lordosis. Front. Bioeng. Biotechnol. 9, 661258. 10.3389/fbioe.2021.661258 34178959 PMC8222614

[B32] Nimmons S. J. B. Simpson A. K. Park A. E. (2020). Decompression alone for the treatment of degenerative lumbar spondylolisthesis. Seminars Spine Surg. 32 (3), 100807. 10.1016/j.semss.2020.100807

[B33] Overdevest G. Vleggeert-Lankamp C. Jacobs W. Thomé C. Gunzburg R. Peul W. (2015). Effectiveness of posterior decompression techniques compared with conventional laminectomy for lumbar stenosis. Eur. Sect. Cerv. Spine Res. Soc. 24 (10), 2244–2263. 10.1007/s00586-015-4098-4 26184719

[B34] Park P. Garton H. J. Gala V. C. Hoff J. T. McGillicuddy J. E. (2004). Adjacent segment disease after lumbar or lumbosacral fusion: review of the literature. Spine 29 (17), 1938–1944. 10.1097/01.brs.0000137069.88904.03 15534420

[B35] Pataky T. C. (2012). One-dimensional statistical parametric mapping in Python. Comput. methods biomechanics Biomed. Eng. 15 (3), 295–301. 10.1080/10255842.2010.527837 21756121

[B36] Pietrantonio A. Trungu S. Famà I. Forcato S. Miscusi M. Raco A. (2019). Long-term clinical outcomes after bilateral laminotomy or total laminectomy for lumbar spinal stenosis: a single-institution experience. Neurosurg. focus 46 (5), E2. 10.3171/2019.2.focus18651 31042648

[B37] Rao R. D. Wang M. Singhal P. McGrady L. M. Rao S. (2002). Intradiscal pressure and kinematic behavior of lumbar spine after bilateral laminotomy and laminectomy. Spine J. official J. North Am. Spine Soc. 2 (5), 320–326. 10.1016/s1529-9430(02)00402-3 14589462

[B38] Raspe H. (2012). Rückenschmerzen. Gesundheitsberichterstattung des Bundes. Berlin, Germany: Robert Koch Institute. 10.25646/3164

[B39] Rihn J. A. Lee J. Y. Khan M. Ulibarri J. A. Tannoury C. Donaldson W. F. (2007). Does lumbar facet fluid detected on magnetic resonance imaging correlate with radiographic instability in patients with degenerative lumbar disease? Spine 32 (14), 1555–1560. 10.1097/brs.0b013e318067dc55 17572627

[B40] Rockenfeller R. Müller A. Damm N. Kosterhon M. Kantelhardt S. R. Frank R. (2020). Muscle-driven and torque-driven centrodes during modeled flexion of individual lumbar spines are disparate. Biomechanics Model. Mechanobiol. 20, 267–279. 10.1007/s10237-020-01382-9 PMC7892748 32939615

[B41] Rohlmann A. Neller S. Claes L. Bergmann G. Wilke H. J. (2001). Influence of a follower load on intradiscal pressure and intersegmental rotation of the lumbar spine. Spine 26 (24), E557–E561. 10.1097/00007632-200112150-00014 11740371

[B42] Rupp T. K. Ehlers W. Karajan N. Günther M. Schmitt S. (2015). A forward dynamics simulation of human lumbar spine flexion predicting the load sharing of intervertebral discs, ligaments, and muscles. Biomechanics Model. Mechanobiol. 14 (5), 1081–1105. 10.1007/s10237-015-0656-2 25653134

[B43] Schär R. T. Kiebach S. Raabe A. Ulrich C. T. (2019). Reoperation rate after microsurgical uni- or bilateral laminotomy for lumbar spinal stenosis with and without low-grade spondylolisthesis: what do preoperative radiographic parameters tell us? Spine 44 (4), E245–E251. 10.1097/brs.0000000000002798 30015718

[B44] Schmidt C. O. Raspe H. Pfingsten M. Hasenbring M. Basler H. D. Eich W. (2007). Back pain in the German adult population: prevalence, severity, and sociodemographic correlates in a multiregional survey. Spine 32 (18), 2005–2011. 10.1097/brs.0b013e318133fad8 17700449

[B45] Schmoelz W. Erhart S. Unger S. Disch A. C. (2012). Biomechanical evaluation of a posterior non-fusion instrumentation of the lumbar spine. Eur. Sect. Cerv. Spine Res. Soc. 21 (5), 939–945. 10.1007/s00586-011-2121-y PMC3337905 22205112

[B46] Silvestros P. Preatoni E. Gill H. S. Gheduzzi S. Hernandez B. A. Holsgrove T. P. (2019). Musculoskeletal modelling of the human cervical spine for the investigation of injury mechanisms during axial impacts. PloS one 14 (5), e0216663. 10.1371/journal.pone.0216663 31071162 PMC6508870

[B47] Steele K. M. Seth A. Hicks J. L. Schwartz M. S. Delp S. L. (2010). Muscle contributions to support and progression during single-limb stance in crouch gait. J. biomechanics 43 (11), 2099–2105. 10.1016/j.jbiomech.2010.04.003 PMC2914221 20493489

[B48] Stokes I. A. Frymoyer J. W. (1987). Segmental motion and instability. Spine 12 (7), 688–691. 10.1097/00007632-198709000-00009 2961083

[B49] Strube P. Putzier M. Siewe J. Eicker S. O. Dreimann M. Zippelius T. (2019). To fuse or not to fuse: a survey among members of the German Spine Society (DWG) regarding lumbar degenerative spondylolisthesis and spinal stenosis. Archives Orthop. trauma Surg. 139 (5), 613–621. 10.1007/s00402-018-3096-5 30542763

[B50] Tai C.-L. Hsieh P.-H. Chen W.-P. Chen L.-H. Chen W.-J. Lai P.-L. (2008). Biomechanical comparison of lumbar spine instability between laminectomy and bilateral laminotomy for spinal stenosis syndrome - an experimental study in porcine model. BMC Musculoskelet. Disord. 9, 84. 10.1186/1471-2474-9-84 18547409 PMC2438358

[B51] Wang K. Deng Z. Chen X. Shao J. Qiu L. Jiang C. (2023). The role of multifidus in the biomechanics of lumbar spine: a musculoskeletal modeling study. Bioeng. (Basel, Switz. 10 (1), 67. 10.3390/bioengineering10010067 PMC9854514 36671639

[B52] Wawrose R. A. LeVasseur C. M. Byrapogu V. K. Dombrowski M. E. Donaldson W. F. Shaw J. D. (2020). *In vivo* changes in adjacent segment kinematics after lumbar decompression and fusion. J. biomechanics 102, 109515. 10.1016/j.jbiomech.2019.109515 31767283

[B53] Wilke H. J. Wolf S. Claes L. E. Arand M. Wiesend A. (1996). Influence of varying muscle forces on lumbar intradiscal pressure: an *in vitro* study. J. Biomech. 29 (4), 549–555. 10.1016/0021-9290(95)00037-2 8964785

[B54] Wiltse L. L. Newman P. H. Macnab I. (1976). Classification of spondylolisis and spondylolisthesis. Clin. Orthop. Relat. Research® 117, 23–29. 10.1097/00003086-197606000-00003 1277669

